# Surgical fixation of hip fractures– a novel technique for pre-operative planning

**DOI:** 10.1186/s13018-025-05803-2

**Published:** 2025-04-19

**Authors:** Zi Qiang Glen Liau, Kamaraj Thirukumaran, Seth Ian Sim, Alexander Shao-Rong Pang

**Affiliations:** 1 Department of Orthopaedic Surgery, National University Hospital, 5 Lower Kent Ridge Rd, Singapore, 119074 Singapore; 2 Department of Orthopaedic Surgery, Alexandra Hospital, 378 Alexandra Rd, 159964 Singapore, Singapore; 3 Yong Loo Lin School of Medicine, National University of Singapore, Singapore, 117597 Singapore

**Keywords:** Femur, Intertrochanteric fractures, Dynamic hip screw fixation, Minimally invasive surgery

## Abstract

**Purpose:**

The dynamic hip screw (DHS) is a widely used method for hip fracture stabilisation, but conventional DHS (CHDS) fixations may be limited by longer surgical duration and delayed recovery compared to minimally invasive DHS (MIDHS) fixations. We describe a novel low-cost surgical method that reduces intraoperative time, peri-operative complications, and improves overall patient outcomes.

**Methods:**

A prospective double-blinded study included 15 patients who underwent surgical fixation of IT hip fractures using a 4-hole DHS system. All surgeries were performed at a tertiary referral hospital between January 2019 and April 2023 by surgeons with similar levels of experience. Main outcome measurements included tip-apex distance (TAD), surgery duration, haemoglobin loss, and hospital stay duration. Two independent assessors measured TAD using the post-operative anteroposterior and lateral radiographs. Kyle’s classification was used to categorize the IT fractures. IBM SPSS Statistics 26.0 for Mac (SPSS, Chicago, IL, USA) was used for the statistical analysis. Statistically significant difference was defined as *p*-value < 0.05.

**Results:**

Both groups had similar baseline characteristics (*p* > 0.05). Both groups had similar complexity in fractures, but the mean surgical duration was significantly shorter (*p* = 0.019) (43.8 ± 12.3 min) compared to the CDHS group (73.4 ± 18.2 min). Postoperatively, there was no significant difference (*p* > 0.05) in hospital stay duration, haemoglobin (Hb) loss, or TAD.

**Conclusions:**

MIDHS group had shorter surgical duration compared to CDHS group, with no significant difference in TAD, haemoglobin loss and hospital stay duration.

## Introduction

### Intertrochanteric hip fractures

Hip fractures are a commonly encountered injury in the field of orthopaedic surgery. Globally, the number of hip fractures is expected to reach 4.5 million by the end of the year 2050, representing a significant increase from 1.26 million cases in 1991 [1, 2]. Asia will account for more than half of all hip fractures, due to its rapidly aging population and higher life expectancy [3]. Hip fractures represent 0.1% (1.75 million disability adjusted life years) of the global burden of disease world-wide [4]. 

Elderly adults are more susceptible to hip fractures as they tend to have reduced mobility and are more likely to have concomitant medical comorbidities [5–7]. In the United Kingdom (UK), after the age of 50, the lifetime risk of hip fracture is approximately 11% for women and 3% for men [8]. Medical comorbidities such as osteoporosis increase the likelihood of a fragility fracture [9–11]. An estimated 5.5 million men and 22 million women in the European Union (EU) alone were estimated to have osteoporosis in 2010. This led to 3.5 million new fragility fractures, including 520,000 vertebral fractures, 610,000 forearm fractures, and 1,800,000 hip fractures [11]. Majority of hip fractures are caused by falls sustained during regular activities, with a male to female ratio of one to four [12, 13]. 

### Minimally invasive dynamic hip screw fixations

The dynamic hip screw (DHS) is a common fixation technique used in the stabilisation of intertrochanteric hip fractures [14–16]. However, conventional dynamic hip screw (CDHS) fixation may be limited by longer surgical duration, more extensive soft tissue dissection, increased blood loss, delayed recovery, and prolonged hospital stays [17, 18]. 

The growing emphasis on optimising patient outcomes and minimisation of surgical trauma has given rise to the development and refinement of minimally invasive dynamic hip screw (MIDHS) fixation. Ideally, a minimally invasive technique should maintain the necessary fixation stability without noticeably shortening the femoral neck or causing rotation or tilting the femoral head [19]. Modification of current surgical techniques have been shown to reduce surgical duration, length of post-operative hospital stays, and blood loss [20, 21]. This prospective study aims to evaluate and compare clinical outcomes of patients who have undergone MIDHS with those who have undergone CHDS.

### Tip-apex distance

**Fig. 1:**
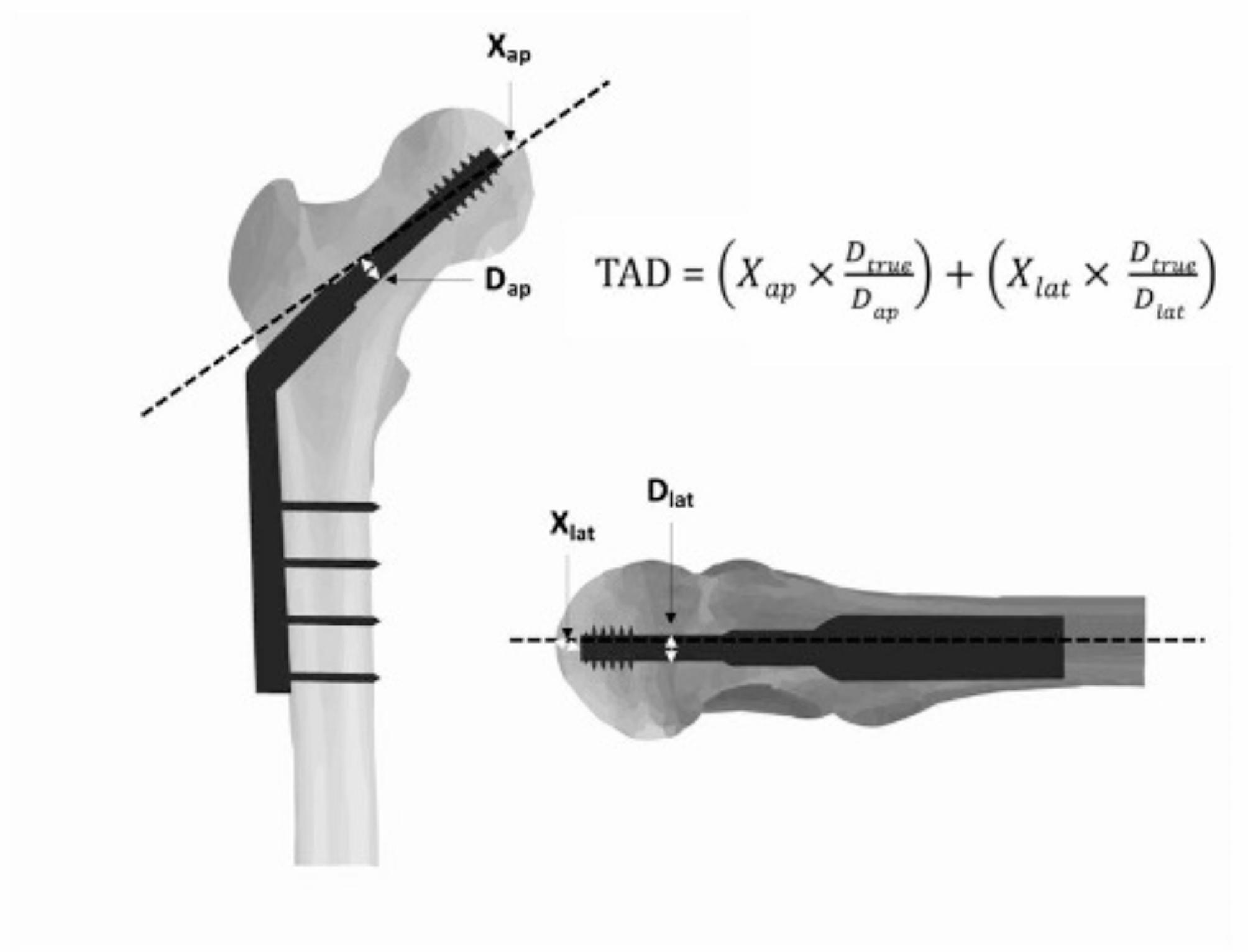
Illustration and formula of Tip-Apex Distance (TAD), adjusted for radiographic magnification [22]– X_ap_ and X_lat_ represent the measured distances shown on the anteroposterior and lateral X-rays, respectively. D_true_ denotes the true diameter of the lag screw, while D_ap_ and D_lat_ refer to the measured diameters of the lag screw as depicted on the anteroposterior and lateral X-rays

Tip-apex distance (TAD) is the total distance measured from anteroposterior and lateral views, between the lag screw tip and the apex of the femoral head [22]. This is illustrated in Fig. 1. For the purpose of the study, the known diameter of the hip screw was used to adjust for radiological magnification.

## Methods

A prospective single-blinded case control study was performed. Patients with intertrochanteric (IT) hip fractures who underwent surgical fixation with 4-hole DHS system in a tertiary referral hospital between January 2019 and April 2023 were identified and evaluated for inclusion in this trial. All cases were performed by Senior Orthopaedic Surgery Residents (Orthopaedic Surgery Registrars) of similar experience levels. Patients with complex fractures requiring open reduction were excluded from the study. The study protocol was approved by the Domain Specific Review Board (DSRB) of the institution under Reference number 2023/00715.

### Novel technique for planning minimally invasive surgical fixation of hip fractures

The authors propose a novel MIDHS technique for treating IT hip fractures that considers the trajectory of the guidewire in all 3 planes– coronal, sagittal and axial, before any incision is made.

Cases were performed either under general or spinal anaesthesia. Prior to starting surgery, patients were positioned on a radiolucent fracture table, and all fractures were successfully reduced by closed manipulation under fluoroscopic control to 10 degrees of valgus on AP radiographs and < 58 degrees of posterior angulation on lateral radiographs. The skin surface markings were performed prior to preparation of the surgical field. The intraoperative imaging that was performed during the drawing of the skin markings were performed with the primary surgeon fully protected and comfortable, without wearing lead gown and thyroid shield yet, by standing behind panelled lead shields and/or outside the theatre leaded doors. The primary surgeon re-positioned the protractor as required in between imaging shots. Only the surgical assistant was required to hold a long rod for the purpose of achieving the lateral femur axis marking in the Clements-Nakayama view [23]. 

#### Antero-posterior surface marking– coronal consideration of plate position

**Fig. 2:**
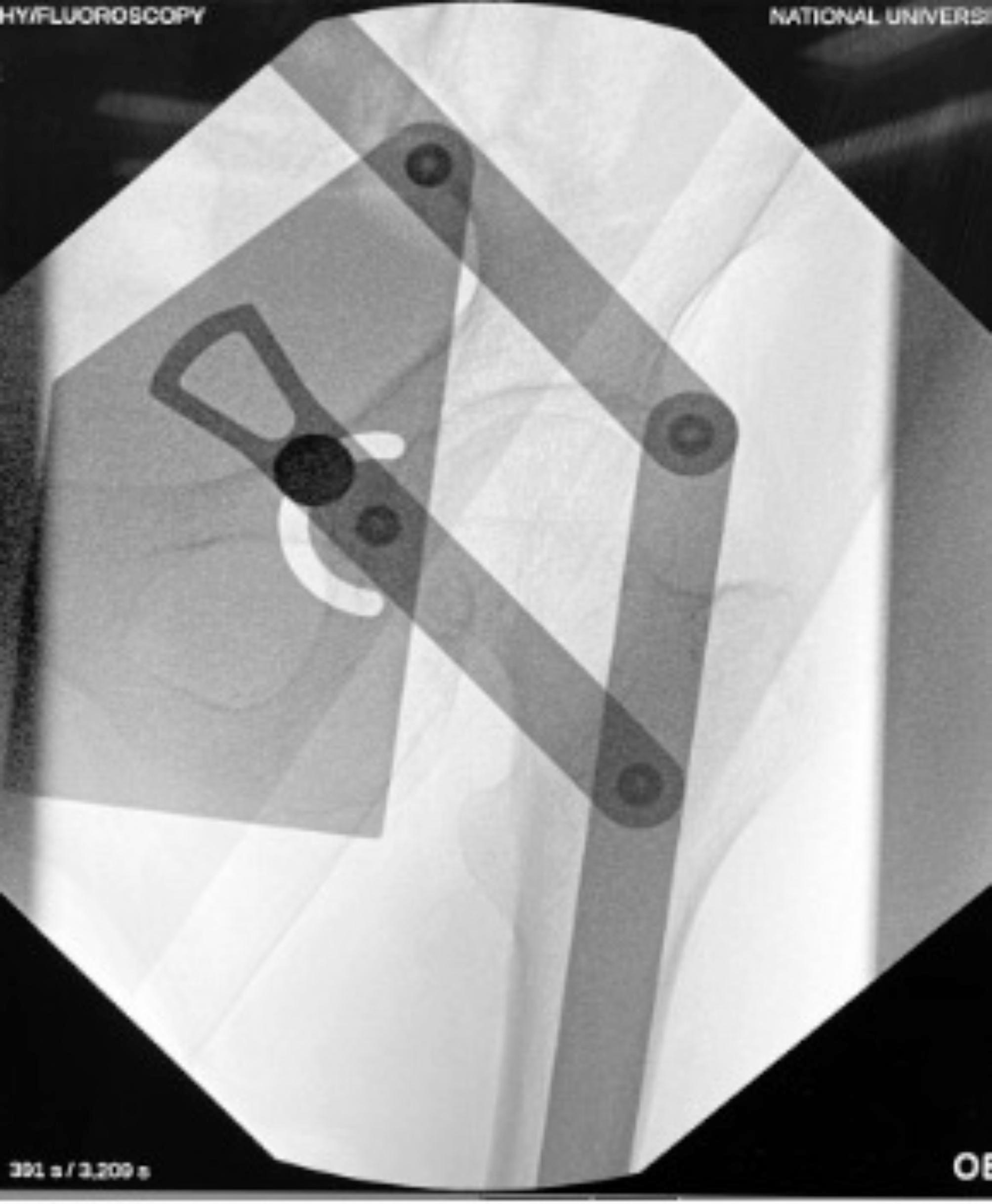
AP view of 135^o^ metal protractor, the vertical limb was overlaid on the patient’s lateral femoral cortex, with the CCD line at the desired height, typically in the lower half of femoral neck and head region

**Fig. 3:**
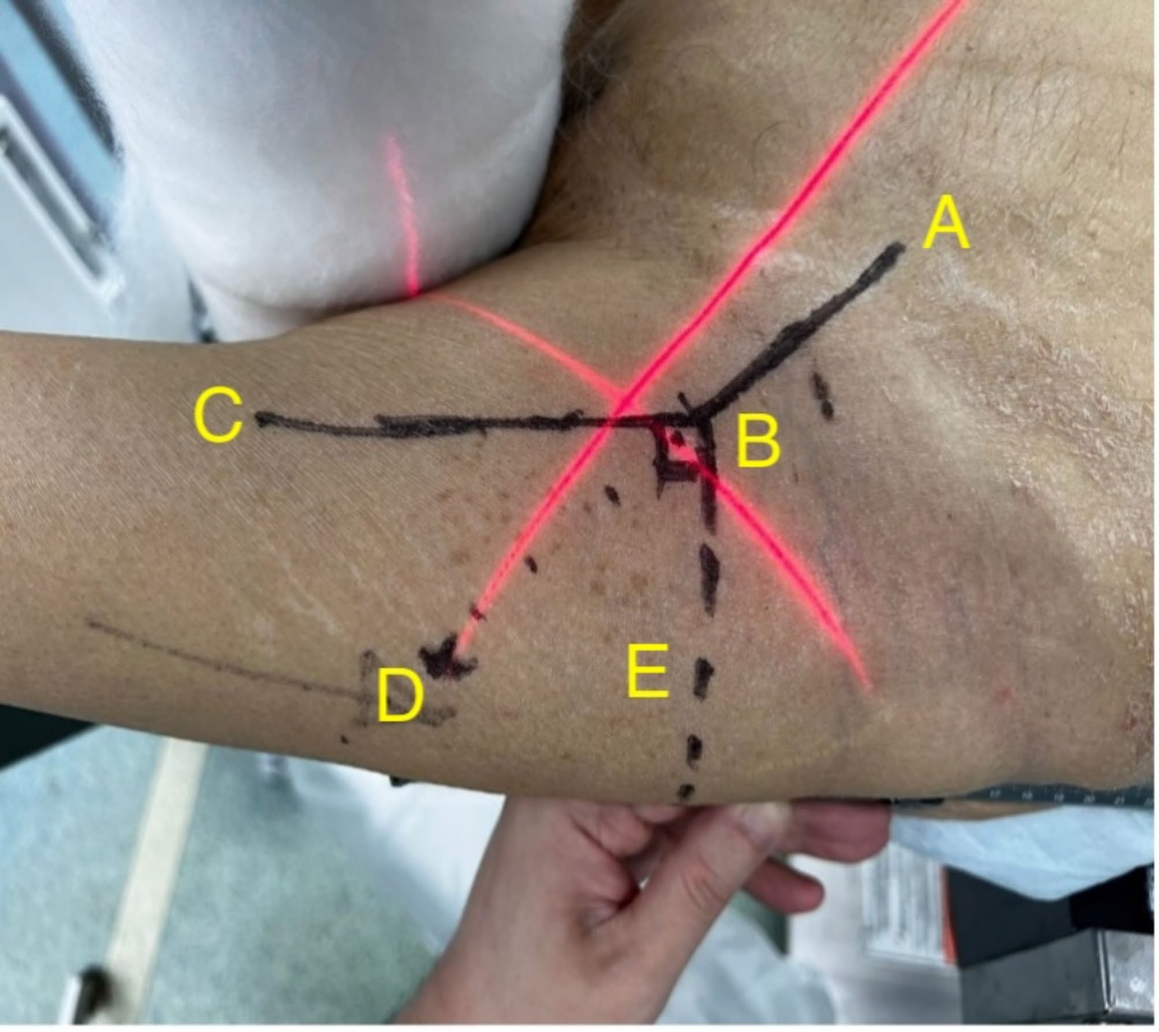
Anterior surface markings– Point A: Line towards centre of femoral head; Point B: Junction of axis of lines A and C; Point C: Along the lateral border of femur; Line D: Extension of line A beyond junction B; Line E: Perpendicular/ vertical line down posteriorly towards the floor

A metal (stainless steel) protractor that was commercially purchased, was adjusted to the desired pre-templated degree– 135 or 150 degrees and laid directly on top of the patient. Antero-posterior (AP) intraoperative imaging (II) X-rays were taken to obtain and mark the surface position of the DHS plate (Figs. 2 and 3). The vertical limb of the protractor was overlaid on the patient’s lateral femoral cortex with the vertical limb overlaid on the patient’s lateral femoral cortex, and the caput-collum-diaphyseal (CCD) line at the desired height, typically in the lower half of femoral neck and head region (Fig. 2). The metallic nature of a protractor provided a satisfactory semi-lucent appearance, overlayed clearly over the femur, on the fluoroscopic image taken.

#### Lateral surface marking– sagittal and axial consideration of plate position

**Fig. 4:**
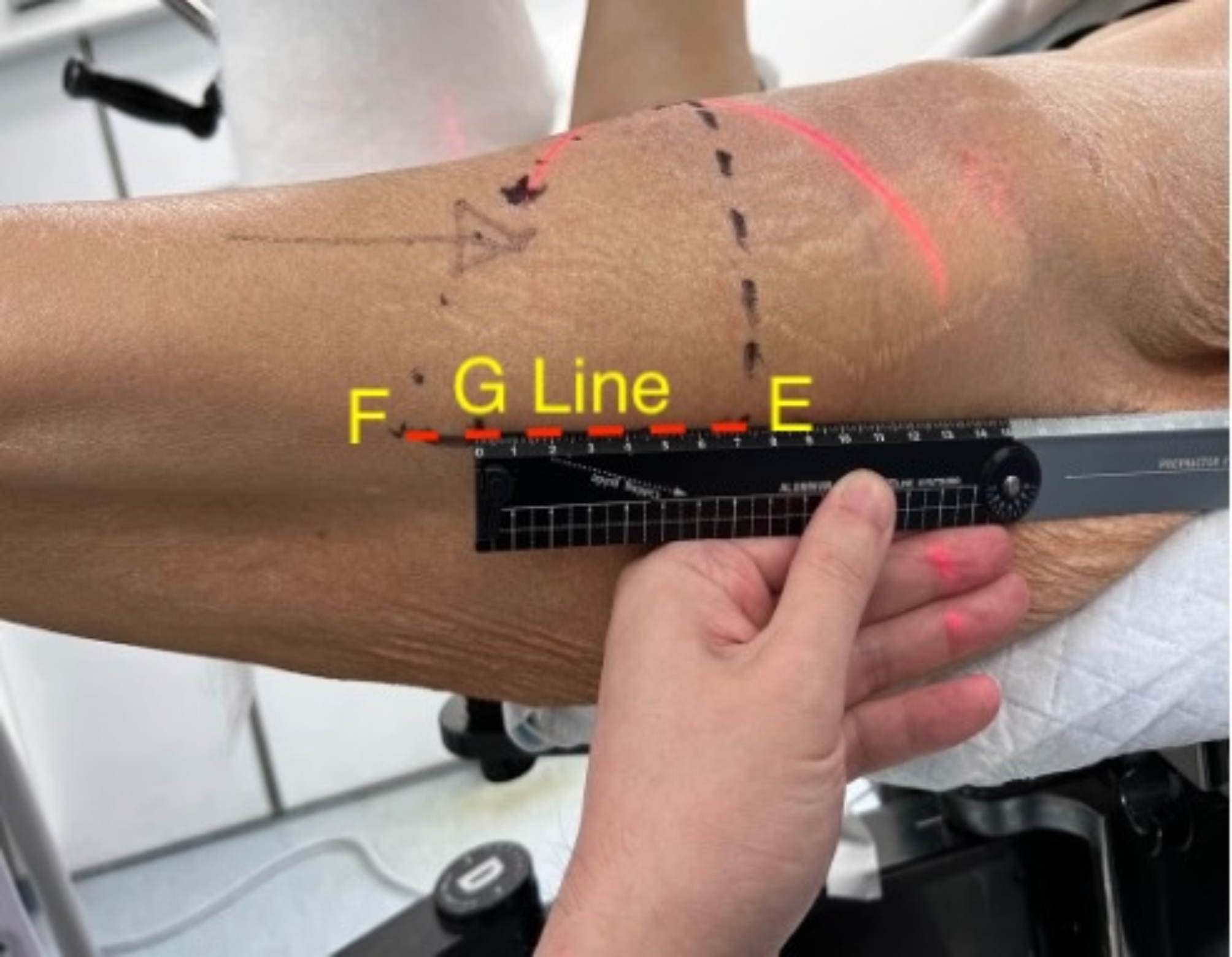
Lateral surface markings– Line E: Perpendicular/ vertical line down towards the floor; Point F: Intersection of lines D and E; Line G: Drawn along the mid axis of femur, referenced from the Clements-Nakayama method

With the Clements-Nakayama method [23]– a modified form of lateral view, resulted in a patient specific view of a collinear femoral head to the femoral shaft, which allowed the surgeon to take into consideration the amount of anteversion the specific patient had. With the II in the Clements-Nakayama tilt, a straight ruler was then used to mark the mid shaft of the femur along this view (Fig. 4).

#### Surface marking– (a) Entry point of guidewire trajectory on skin surface (b) Length of potential incision

- Point B, the intersection of the extrapolation of the oblique CCD line distally to meet the lateral surface marking line, was the entry point of the guidewire trajectory on the skin surface (Fig. 3). This accounted for the thickness of the subcutaneous tissues and musculature of the specific patient.
- Line E, a perpendicular line from the junction of the vertical limb and CCD angle **(**Figs. 3 and 4**)**, was made posteriorly, to meet the G line, which was the lateral surface marking line (Fig. 4).

The line between the start of line E and point F, the G line, was the length of the potential skin incision to be made (Fig. 4). In practice, depending on the thickness, and the turgor of the patient’s soft tissues, this line can be shortened up to 1–2 cm proximally, and 2–4 cm distally, and retractors may be used to allow for implant insertion.

Skin preparation was then done carefully, to avoid erasure of the markings during cleansing. Intraoperatively, after skin incision and exposure, the surgeon may additionally use a free K-wire, and insert it freehand, anterior to the cortex of the femur, to end in the femoral head. This served as a secondary visual guide for the anteversion of the guidewire to be inserted.

This abovementioned method enables surgeons to ascertain a more precise location and trajectory of the guidewire, starting from the entry point on the skin. This allows for a potentially smaller minimally invasive surgical incision. By accurately placing the guidewire with confidence on the first try, it allows surgeons to know the exact distal length of incision required for the DHS plate leading to reduction in soft tissue dissection.

### Pre-operative parameters

Pre-operative clinical data for both groups is presented in Table 1. Participants’ age, gender, body mass index (BMI), and premorbid ambulatory status were recorded. Participants’ comorbidities were summarised into a score using the Charlson comorbidity index, alongside an estimated 10-year survival rate which was calculated.

**Table 1:** Pre-operative data for both groups

Variable	MIDHS (95% CI), *n* = 5	CDHS (95% CI), *n* = 10	*p*-value
Age, *M*^#^	75 (73.5, 80.5)	80 (69.3, 82.7)	0.322
Gender^^^			
Men: Women	1:4	4:6	0.600
Body mass index (kg/m2), *M* ± S.D. (R) ^#^	22.4 ± 5.1	22.9 ± 3.4	0.354
Charlson comorbidity index, *n*, *M* ± S.D. (R) ^#^	4.2 ± 1.6	2.5 ± 3.5	0.371
Estimated 10-year survival rate (%), *M* ± S.D. (R) ^#^	52 ± 31.4	33.0 ± 40.0	0.440
Premorbid ambulatory status, *n*^^^			
Unaided	2	5	1.00
Walking stick	1	2	
Walking frame	1	1	
Wheelchair	1	1	
Homebound	0	1	

MIDHS, minimally invasive dynamic hip screw (fixation); CDHS, conventional dynamic hip screw (fixation); *M*, mean; S.D., standard deviation; R, range; *n*, number of participants; Hb, haemoglobin; ASA, American Society of Anaesthesiologists’ classification of physical status

*Statistically significant difference, *p* < 0.05

^#^ Variables analysed using the Mann-Whitney U test

^^^ Variables analysed using the Fisher’s exact test

### Surgical outcome assessment

Post-operatively, patients received the same treatment and standard rehabilitation protocol established at the tertiary referral hospital. Surgical duration was defined as knife to skin, to completion of skin closure. All patients had their wounds closed in layers, with sutures only - no staplers were used. In all cases, AP and lateral radiographs were obtained post-operatively to assess fracture fixation and implant positioning. Pre- and post-operative haemoglobin levels were measured to assess the amount of blood loss. Two independent assessors measured the TAD using the post-operative AP and lateral radiographs. Discrepancies of > 2 mm between the measurements by the two assessors were identified and remeasured. The Kyle’s classification was used to group the IT fractures into four categories– 1, stable 2-part IT fractures without displacement and tearing; 2, stable 3-part IT fractures with displacement and minimal tearing; 3, unstable 4-part IT fractures with displacement and posterior-medial breakage; 4, unstable 4-part IT fractures with posterior displacement, posterior-medial breakage and inferior-trochanterion component [24]. 

### Statistical analysis

IBM SPSS Statistics 26.0 for Mac (SPSS, Chicago, IL, USA) was used for the statistical analysis. Analysis of the categorical variables (Kyle’s classification and ASA classification) was performed using the Chi square test since *n* > 5. Analysis of the continuous variables comprising of non-parametric data (Tip-apex distance, duration of surgery, days between injury and surgery, days of hospital stay, Hb loss, number of intraoperative radiographs and cumulative duration of radiation exposure) was performed using the Mann-Whitney U test. 95% confidence interval was used to provide us with the likely values of the true population mean. The significance tests for each were all two-tailed. Statistically significant difference was defined as *p*-value < 0.05.

## Results

15 patients met the inclusion criteria and were sorted accordingly into the MIDHS or CDHS group. All MIDHS surgeries were performed by a single surgeon, a second- to third-year Senior Orthopaedic Resident (Orthopaedic Surgery Registrar). Other surgeons with similar experience performed CDHS fixation on the control group. Both groups utilised the same implants, sets and tools. The pre- and post-operative clinical information were documented for each case.

The MIDHS and CDHS groups comprised 5 and 10 patients respectively. Both groups had comparable preoperative demographics, body mass index, comorbidity factors (Charlson Comorbidity index), premorbid ambulatory status, injury mechanism, fracture pattern and time elapsed from injury to surgery. The American Society of Anaesthesiologists (ASA) classification for both groups was also similar. The ASA classification for one patient was not recorded and was excluded from the study (Table 1). MIDHS group had a similar complexity of fractures compared to the CDHS group (*p* = 0.097). However, the mean surgical duration in the MIDHS group was significantly shorter as compared to the CDHS group (43.7 vs. 73.4 min, *p* = 0.019).

No significant difference was found for the following outcome measures: duration of hospital stays (*p* = 0.310), TAD (*p* = 0.594), haemoglobin (Hb) loss (*p* = 0.898), number of intraoperative radiographs (*p* = 0.825) and cumulative duration of radiation exposure (*p* = 0.604). The surgical and post-surgical data for both groups is summarised in Table 2. Lower mean TAD, Hb loss and duration of hospital stay were observed in the MIDHS group, but the outcomes were not statistically significant.

**Table 2:** Surgical and Post-Surgical data for both groups

Variable	MIDHS (95% CI), *n* = 5	CDHS (95% CI), *n* = 10	*p*-value
Tip-apex distance (mm), *M* ± S.D. (R)^#^	12.6 ± 3.88, 95% CI [9.2, 16.0]	14.5 ± 5.23, 95% CI [11.3, 17.8]	0.594
Duration of surgery (min), *M* ± S.D. (R) ^#^	43.8 ± 12.26, 95% CI [33.0, 54.6]	73.4 ± 18.2, 95% CI [62.1, 84.7]	0.019*
Days between injury and surgery, *M* ± S.D. (R) ^#^	3.4 ± 4.28, 95% CI [-0.4, 7.2]	1.9 ± 0.7, 95% CI [1.4, 2.4]	0.953
Days of hospital stay, *M* ± S.D. (R)^#^	8.4 ± 5.5, 95% CI [3.6, 13.2]	9.8 ± 4.5, 95% CI [7.0, 12.6]	0.310
Kyle’s classification, *n*^*^*^ I II III IV	0 3 1 1	6 3 1 0	0.097
ASA classification, *n*^*^*^ I II III IV	0 3 1 0	0 6 4 0	1.00
Hb loss (g/dl), *M ±* S.D. (R)^#^	1.9 ± 1.1, 95% CI [1.0, 2.8]	2.1 ± 1.8, 95% CI [1.0, 3.2]	0.898
Number of intraoperative radiographs^#^	91.3 [73.6,108.9]	97.7 [84.7, 110.7]	0.825
Cumulative duration of radiation exposure^#^	72.0 [54.7, 89.3]	70.9 [63.2, 78.6]	0.604

MIDHS, minimally invasive dynamic hip screw (fixation); CDHS, conventional dynamic hip screw (fixation); *M*, mean; S.D., standard deviation; R, range; *n*, number of participants; Hb, haemoglobin; ASA, American Society of Anaesthesiologists’ classification of physical status

*Statistically significant difference, *p* < 0.05

^#^ Variables analysed using the Mann-Whitney U test

^^^ Variables analysed using the Fisher’s exact test

## Discussion

Several methods and techniques have been described for the surgical treatment of IT fractures [25–27]. DHS is commonly used for treating IT fractures in the elderly as it allows for early remobilisation, is easy to use, and has low complication rates [28, 29]. However, it is imperative to note that certain parameters such as patient’s age, the amount of time elapsed between the injury and surgery, and the presence of medical comorbidities also play a role in treatment outcomes [30]. Several studies have explored MIDHS fixations with favourable clinical outcomes. However, there are various limitations can be identified in the existing literature.

A similar study conducted by Wong et al. [21], the MIDHS group had considerably smaller drops in Hb levels, lower rate of blood transfusions, significantly lower pain scores on the third postoperative day, and significantly lesser overall analgesic use in the first three days. However, Wong’s MIDHS method showed no significant difference in surgical duration. Our MIDHS method records lower mean surgical duration in surgeons of similar surgical experience, suggesting that it may be a superior technique. This may be due to the additional consideration of the anteversion of the femur, with the incorporation of the Clements-Nakayama view. Although Wong’s MIDHS group average hospital stay was shorter than that of the CDHS group, neither the difference in complication rates nor the difference in hospital stay duration were statistically significant.

Ho et al. [31] described a minimally invasive technique with significant shortening in the duration of surgery and length of hospital stay. However, the paper considered 2-hole, 3-hole, and 4-hole DHS and is not fully relevant to our current study which only studies the use of a 4-hole DHS system. Furthermore, the TAD and Hb loss were also reported to be statistically insignificant as well. This is concurrent with our findings.

Lee et al. [18] reported positive findings with MIDHS with statistically significant haemoglobin loss and length of hospital stay which is inconsistent with our data. However, unlike our novel technique, this study did not prove a statistically significant reduction in surgical duration. Furthermore, they preferred the use of 3-hole side plates, whereas this study used 4-hole side plates. Thus, a direct comparison to their study cannot be made.

Wang et al. [32] reports a MIDHS technique with significantly lower average surgical duration, incision length, blood transfusion rate, and post-operative stay. While Wang had a significantly shorter mean surgical time in the MIDHS group, the mean of 64.8 min was much higher than that of ours, with a mean of 43.8 min.

Our MIDHS group also showed superior outcomes in mean TAD, haemoglobin loss, and length of hospital stay, though they were not statistically significant. This shows that our novel technique is indeed an improvement to existing surgical techniques and increases the overall standard of care. While our mean TAD is better, there is no consensus in literature regarding the minimum clinically important difference (MCID) with regards to TAD.

### Limitations

Although the results proved that the proposed technique produced better results as compared to CDHS, there were several limitations of the study as well as the novel MIDHS technique. Firstly, the statistical power of the study was reduced given a small sample size (*n* = 15). The surgical technique was conducted by a single surgeon, and hence the reproducibility of the results must be validated by other surgeons of the same level of experience. Although a larger sample size would be ideal, the surgeon has shown that the duration of surgery was clinically and statistically significantly shorter as compared to the CDHS. Although more intraoperative images were taken pre-incision using our novel method, this allowed for fewer intraoperative images, and fewer total images to be taken after skin incision. This reduced wound exposure time, blood loss, and attempts for entry of the guidewire.

A further limitation for both the MIDHS and CDHS groups was the lack of recorded data on the number of repeated attempts needed to achieve satisfactory guidewire placement. However, in the author’s experience, a maximum of two to three passes with minimal adjustment were needed to reach the optimal position, due to improved accuracy from the surface markings. The Kyle screw position was not examined in this study. However, MIDHS had a lower TAD as compared to the CDHS control group, which was the most important independent factor when assessing for the optimal positioning of the DHS. All patients included in the study reached union with appropriate TAD targets of < 25 mm achieved. Lastly, the Harris hip score and Elderly mobility scale, which assess long term rehabilitative progress of surgical patients, were not included as outcome measurements.

## Conclusion

Minimally invasive surgeries are known to reduce post-operative complications and morbidity. Our MIDHS technique proved that in comparison to the CDHS, there was no significant difference in hospital stay duration, haemoglobin loss or TAD. However, there was a significant reduction in surgical duration. Furthermore, the number of passes before guidewire positioning could affect the clinical outcome of the patient and therefore the reliability of the conclusion. This suggests that to ascertain and validate our study further, we need to increase our cohort size, include the number of passes made in both case series, and follow up with our patients over the long term.

## Data Availability

No datasets were generated or analysed during the current study.

## Abbreviations

AP: Antero-posterior
BMI: Body mass index
CCD: Caput-collum-diaphyseal
CDHS: Conventional dynamic hip screw
DHS: Dynamic hip screw
DSRB: Domain Specific Review Board
EU: European Union
Hb: Haemoglobin
IT: Inter-trochanteric
MCID: Minimum clinically important difference
MIDHS: Minimally invasive dynamic hip screw
TAD: Tip-apex distance
UK: United Kingdom
